# DL-3n-Butylphthalide Improves Blood–Brain Barrier Integrity in Rat After Middle Cerebral Artery Occlusion

**DOI:** 10.3389/fncel.2020.610714

**Published:** 2021-01-12

**Authors:** Muyassar Mamtilahun, Zhenyu Wei, Chuan Qin, Yongting Wang, Yaohui Tang, Fan-xia Shen, Heng-Li Tian, Zhijun Zhang, Guo-Yuan Yang

**Affiliations:** ^1^Shanghai Jiao Tong University Affiliated Sixth People's Hospital, School of Biomedical Engineering, Shanghai Jiao Tong University, Shanghai, China; ^2^University of Shanghai for Science and Technology Affiliated Shidong Hospital, Shanghai, China; ^3^Department of Neurology, Ruijin Hospital, School of Medicine, Shanghai Jiao Tong University, Shanghai, China; ^4^Department of Neurosurgery, Shanghai Jiao Tong University Affiliated Sixth People's Hospital, Shanghai Jiao Tong University, Shanghai, China

**Keywords:** AQP4, blood-brain barrier, dl-3n-butylphthalide, edema, ischemic stroke

## Abstract

**Objective:** DL-3n-butylphthalide (NBP) has beneficial effects in different stages of ischemic stroke. Our previous studies have demonstrated that NBP promoted angiogenesis in the perifocal region of the ischemic brain. However, the molecular mechanism of NBP for blood–brain barrier protection in acute ischemic stroke was unclear. Here, we explored the neuroprotective effects of NBP on blood–brain barrier integrity in the acute phase of ischemic stroke in a rat model.

**Methods:** Adult male Sprague–Dawley rats (*n* = 82) underwent 2 h of transient middle cerebral artery occlusion and received 90 mg/kg of NBP for 3 days. Brain edema, infarct volume, surface blood flow, and neurological severity score were evaluated. Blood–brain barrier integrity was evaluated by Evans blue leakage and changes in tight junction proteins. We further examined AQP4 and eNOS expression, MMP-9 enzyme activity, and possible signaling pathways for the role of NBP after ischemic stroke.

**Results:** NBP treatment significantly increased eNOS expression and surface blood flow in the brain, reduced brain edema and infarct volume, and improved neurological severity score compared to the control group (*p* < 0.05). Furthermore, NBP attenuated Evans blue and IgG leakage and increased tight junction protein expression compared to the control after 1 and 3 days of ischemic stroke (*p* < 0.05). Finally, NBP decreased AQP4 expression, MMP-9 enzyme activity, and increased MAPK expression during acute ischemic stroke.

**Conclusion:** NBP protected blood–brain barrier integrity and attenuated brain injury in the acute phase of ischemic stroke by decreasing AQP4 expression and MMP-9 enzyme activity. The MAPK signaling pathway may be associated in this process.

## Introduction

Stroke is one of the leading causes of death and disability worldwide (Feigin et al., 2017). Due to a narrow treatment window, complex pathology, and limited treatment options, stroke ultimately causes patient disability or death, proving to be an enormous economic burden to families and society (Writing Group et al., 2016; Feigin et al., 2017). Approximately 87% of stroke cases are ischemic strokes in the clinic; from the moment cerebral vascular occlusion occurs, the brain tissue suffers a series of pathological cascades including energy failure, increase in reactive oxygen species (ROS), free radical formation, inflammation, and neuronal apoptosis, which could last for several days (Kalogeris et al., 2016). These pathological processes upregulate aquaporin 4 (AQP4) expression and enhance matrix metalloproteinase-9 (MMP-9) enzyme activity. Both AQP4 and MMP-9 increase water uptake and degradation of protein in the blood–brain barrier (BBB) tight junction, which is the main cause of brain edema (Ribeiro et al., 2006; Lakhan et al., 2013; Turner and Sharp, 2016). Brain edema leads to cerebral parenchymal swelling, increased intracranial pressure, and decreased blood flow (Rosenberg, 1999; Mamtilahun et al., 2019). Without medical treatment, edema can result in secondary brain injury and aggravate stroke prognosis.

In the normal brain, the BBB facilitates brain homeostasis by separating the brain tissues from the peripheral circulation. BBB disruption is a canonical pathological characteristic in the acute stage of stroke. It not only causes brain edema but also allows the passage of phagocytes, red blood cells, and metabolic products that induce inflammatory responses and neuronal cell death, which together contribute to the high mortality of ischemic stroke (Yang and Rosenberg Gary, 2011; Obermeier et al., 2013). Hence, targeting BBB protection may be a viable approach for acute ischemic stroke therapy.

Dl-3n-butylphthalide (NBP) is a synthesized drug that was first extracted from *Apium graveolens* Linn seeds (celery) and approved for clinical usage in China by the National Medical Products Administration of China in 2002 (Wang et al., 2018). Clinical and experimental studies have shown that NBP attenuates cerebral infarct size and neurobehavioral deficiency through multitargeted effects, including antiplatelet aggregation (Wang et al., 2016), improvement of mitochondrial functions, antithrombosis (Qin et al., 2019), and reduction of both neurovascular inflammation (Yang et al., 2019) and apoptosis (Zhang et al., 2010). Previously, we found that NBP treatment promoted middle cerebral artery (MCA) dilation and increased microvessel density in ischemic rats (Qin et al., 2019; Zhou et al., 2019). NBP was approved for acute ischemic stroke stage II clinical studies by the Food and Drug Administration (FDA) in 2016 (Wang et al., 2018). Even though there are extensive studies on the therapeutic effects of NBP in ischemic stroke, more robust evidence regarding its effects and underlying mechanisms is required to recommend universal clinical use. Particularly, the neuroprotective effects of NBP on BBB integrity and its underlying mechanism remain unclear. In the present study, we explored the effect of NBP on the functional and structural integrity of the BBB, and whether AQP4 and MMP-9 are involved in this process.

## Materials and Methods

### Experimental Design

**Figure 1:**
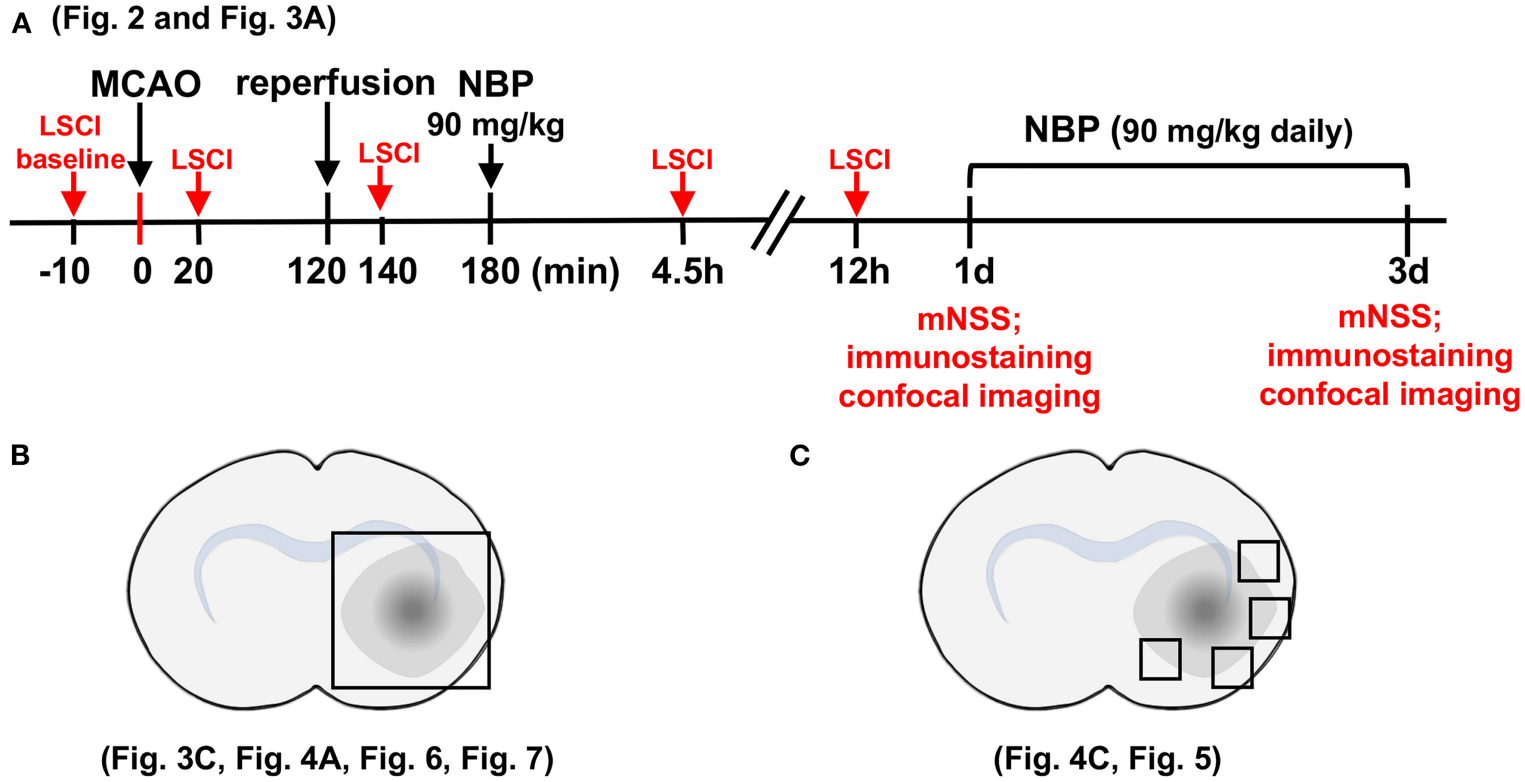
Schematic for experimental design and brain regions used for immunofluorescence analysis. **(A)** Graph illustrates the experimental design and sample collection schedule. Male SD rats underwent transient middle cerebral artery occlusion (tMCAO) for 2 h; 90 mg/kg of dl-3n-butylphthalide (NBP) or vehicle was orally administered at 3 h after MCAO (1 h after reperfusion) for the first time, and then twice daily. The modified neurological severity scores (mNSS) scores were evaluated on day 1 and day 3 after tMCAO. **(B)** Photographs illustrate the brain samples collected after the neurobehavioral tests to evaluate gap formation in the blood–brain barrier (BBB) tight junction proteins. **(C)** Photographs illustrate brain regions used for immunostaining and analysis.

The rat brain surgery procedure and experimental protocol were approved by the Institutional Animal Care and Use Committee (IACUC) of Shanghai Jiao Tong University, Shanghai, China. Adult male Sprague–Dawley (SD) rats (*n* = 82, Jiesijie Laboratory Animal Co., Shanghai, China), weighing 250–280 g, were used in this study. All rats were kept in a humidity-controlled house at 22–25°C with 12-h dark/light cycling, and were allowed to eat and drink freely. The SD rats underwent 2 h of transient middle cerebral artery occlusion (tMCAO) and were randomly assigned to NBP or vehicle (vegetable oil) treatment by daily oral gavage for 3 consecutive days. Cerebral blood flow was assessed using laser speckle contrast imaging (LSCI) at the five indicated time points (Figure 1A). Neurobehavioral outcomes of tMCAO rats were evaluated by neurological severity score (NSS) on days 1 and 3 after tMCAO, following which, rats were euthanized for further immunohistochemistry analysis. For Western blot and Evans blue analysis, the samples were collected from the indicated parts of the brain (Figure 1B). For immunostaining, five to seven animals in each group, with four brain sections from each animal were used. Next, four peri-infarct areas of each brain section were randomly chosen to take confocal images (Figure 1C).

### Surgical Procedure for Transient Middle Cerebral Artery Occlusion

Ischemic brain injury was induced by tMCAO, as described in our previous studies (Li et al., 2013). Briefly, the rat was anesthetized with 4% isoflurane, and anesthesia was maintained with 2% isoflurane during the entire surgical procedure. The body temperature was maintained at 37 ± 0.5°C using a heating pad (RWD Life Science Co., Shenzhen, China). The left common carotid, internal carotid, and external carotid arteries were carefully isolated. A silicon coded 4-0 round top nylon suture (Covidien, Mansfield, MA, USA) was carefully inserted from the left external carotid artery into the internal carotid artery and the origin of the middle cerebral artery. The surface cerebral blood flow (CBF) was monitored, and a decrease to 80% of the baseline value was considered as MCA occlusion. Reperfusion was performed using a suture draw after 2 h of MCAO. To ensure reproducibility of the tMCAO model, the surface blood flow was monitored before and after MCAO, and after reperfusion. Blood oxygen (*P*O_2_), blood carbon dioxide (*P*CO_2_), blood pH, glucose (Glu), sodium (Na), and potassium (K) were determined using an i-STAT system (Abbott Point of Care Inc., Princeton, NJ, USA). NBP (purity > 99.5%) was obtained as a gift from Shijiazhuang Pharmaceutical Group Co. Ltd., China, and dissolved in vegetable oil. tMCAO rats were randomly divided into two groups: NBP treated and control group. NBP (90 mg/kg) or equal volume of vegetable oil was orally administered 3 h after MCAO for the first-time treatment, and then twice daily until sacrificing the animals. MAPK signaling inhibitor PD98059 (10 mg/kg, Meilun, Dalian, China) were tail intravenously injected to rats 1 h before tMCAO as per manufacturers' instruction.

### Evaluation of Neurological Severity Score

At 1 and 3 days after tMCAO, modified NSS (mNSS) was examined to evaluate animal neurological status including sensory, motor, reflex, and balance tests (Tang et al., 2014). The normal mNSS was 0, and the maximal deficit score was 14 (Li et al., 2000). Rats were raised by the tail, and the flexion of the forelimb was tested for motor function evaluation (0–3). Rats' gait was examined by placing them on the floor (0–3). For the beam balance test, rats were placed on a 60-cm-long beam, and their posture was examined (0–6). The sensory tests (0–2) for touch-reflex were used to assess the pinna and corneal reflex.

### Laser Speckle Contrast Imaging

LSCI was performed using a high-resolution laser speckle contrast imaging system (LSCI-2 system, Dolphin BioTech Ltd., Shanghai, China). The imaging protocol has been described previously (Lin et al., 2013). After anesthetizing the rats, a midline incision was made on the scalp, and the skull was exposed. A dental drill was used to remove the surface of the skull until surface blood vessels were detected beneath the skull. Raw speckle images (696 × 512 pixels, 40 μm/pixel) were acquired at 23 fps (exposure time *T* = 5 ms) under 780 nm laser illumination. In each measurement, 20 consecutive frames of speckle images were detected and recorded. Image processing was carried out offline using MatLab software. In order to reduce the noise, the raw LSCI images were aligned using the registered laser speckle contrast analysis method (Wang et al., 2019). The registered speckle images were then analyzed using a random process estimator method to detect the contrast image with improved signal-to-noise ratio (Miao et al., 2010). Relative blood flow was calculated as described in our previous study (Lin et al., 2013). The CBF was detected using LSCI before MCAO, 10 min after occlusion, 20 min after reperfusion, and 4.5 and 12 h after occlusion in NBP-treated and control rats.

### Assessment of Brain Edema and Ischemic Infarct Volume

Frozen brain sections 20 μm in thickness from the anterior commissure to the hippocampus were collected for immunohistochemistry. Serial frozen sections, 20 μm in thickness and 200 μm in interval from the frontal cortex were stained with 0.1% cresyl violet (Meilun Chemical Reagent Co., Dalian, China). Infarct volume was determined by subtracting the area stained with cresyl violet in the ipsilateral hemisphere from that of the contralateral hemisphere using the Image J software (NIH, Bethesda, MD, USA, RRID: SCR_003070), and then multiplying by section interval thickness.

$$\upsilon =\sum_{1}^{\text{n}}[\left({\text{s}}_{\text{n}}+\sqrt{{\text{s}}_{\text{n}}\times {\text{s}}_{\text{n}+1}}+{\text{s}}_{\text{n}+1}\right)\times \frac{\text{h}}{3}]$$

The *h* = 0.2 mm represents the distance between each section, and S represents the area (mm^2^) in each brain section.

### Determining Permeability of the Blood-Brain Barrier

Rats were sacrificed after 1 and 3 days of tMCAO using a high dose of chloral hydrate (10%) anesthesia. The extravasation of Evans Blue (EB, Sigma-Aldrich, St. Louis, MO, USA) and IgG were used to assess BBB permeability. EB dye solution (2% EB dye in saline, 4 ml/kg) was injected through the left jugular vein at 4.5 h, 1 day, and 3 days after tMCAO (Tang et al., 2014). The rats were sacrificed via cardiac perfusion after 2 h of EB circulation under anesthetized conditions. Both brain hemispheres were weighed, and the samples were homogenized in 1 ml of 50% trichloroacetic acid solution to extract EB, followed by centrifugation at 12,000 g for 20 min. The supernatant was diluted with 100% ethanol at a ratio of 1:3. The amount of EB was quantified at 610 nm using a spectrophotometer (Bio-Tek, Winooski, VT).

IgG was examined as previously described (Tanno et al., 1992; Tang et al., 2014). Briefly, brain slices were fixed in 4% paraformaldehyde (PFA) solution, blocked in 10% bovine serum albumin (BSA), incubated in biotinylated antibody for 30 min at room temperature, and incubated in Avidin: Biotinylated Enzyme Complex (ABC) reagent (Vector Labs, Burlingame, CA, USA) for 30 min. DAB staining was used for the visualization of immune reactivity (Vector Labs, Burlingame, CA, USA), and the sections were counterstained with hematoxylin. We randomly chose four fields from the area of interest in each section and used the Image J software (NIH, Bethesda, MD, RRID: SCR_003070) for mean integrated optical density (IOD) analysis.

### Immunohistochemistry

Brain sections were fixed for 10 min in cold methanol at 4°C, then blocked with 10% BSA for 1 h at room temperature after washing with PBS thrice. The brain sections were then incubated with antibodies against occludin (1:100, Invitrogen Cat# 33-1500, RRID: AB_87033), zonula occludens-1 (ZO-1, 1:100, Invitrogen Cat# 61-7300, RRID: AB_138452), and CD-31 (1:200, R&D Systems Cat# AF3628, RRID: AB_2161028) overnight at 4°C. The sections were stained with fluorescence-conjugated secondary antibodies for 1 h at 37°C after washing with PBS thrice. The gap length was shown as a percentage (%) of the whole tight junction staining. Similarly, four brain sections were stained for each animal, and four fields were randomly selected from each brain section (upper, middle, and bottom of the peri-infarct region), using confocal microscopy. Data were analyzed using the Image J software (NIH, Bethesda, MD, USA, RRID: SCR_003070).

### Western Blot Analysis

At 1 and 3 days after tMCAO, fresh rat brains were collected and sectioned into 2-mm slices that included the ischemic core and peri-infarct areas in a rat brain mold (RWD Company, Shenzhen, China), and peri-infarct areas were collected from the ipsilateral cerebral hemisphere for the Western blotting experiments. Homogenizing buffer (RIPA with protease cocktail inhibitor, phosphatase inhibitor, and phenylmethanesulfonyl fluoride) was used for the brain sample collection. The homogenate was centrifuged at 12,000 *g*, and the pellets were discarded. Protein concentration was examined using a BCA kit (Meilun, Dalian, China), and 40 μg of protein from each group was loaded onto 10% resolving gel for electrophoresis. Then protein was transferred to a nitrocellulose membrane (GE Healthcare Life Sciences, Pittsburgh, PA, USA), and 5% skim milk was used for blocking. Next, the membranes were incubated with primary antibodies against AQP4 (1:1,000, Santa Cruz Biotechnology Cat# sc-58612, RRID:AB_781471), occludin (1:1,000, Invitrogen Cat# 33-1500, RRID: AB_87033), zonula occludens-1 (ZO-1, 1:1,000, Invitrogen Cat# 61-7300, RRID: AB_138452), eNOS (1:1,000, BD Biosciences Cat# 610298, RRID:AB_397692), phospho-MAPK (1:1,000, Cell Signaling Technology Cat# 4370, RRID:AB_2315112), MAPK (1:1,000, Cell Signaling Technology Cat# 4695, RRID:AB_390779), and β-actin (1:1,000, Santa Cruz Biotechnology Cat# sc-47778 HRP, RRID:AB_2714189) overnight at 4°C. HRP-conjugated secondary antibody and enhanced chemiluminescence substrate (Pierce, Rockford, IL, USA, www.piercenet.com) were used for visualization after washing thrice with TBST buffer. The results of chemiluminescence were assessed with an imaging system (Bio-Rad, Hercules, CA, USA, www.bio-rad.com). The relative levels of AQP4, occludin, ZO-1, and eNOS were normalized to that of β-actin. The pMAPK levels were normalized to those of total MAPK.

### Real-Time PCR Analysis

Total RNA from brain tissue samples was isolated using TRIzol Reagent (Life Technologies). RNA concentration was examined using a spectrophotometer (NanoDrop 1000, Thermo Fisher) followed by a reverse transcription process using the PrimeScript RT reagent kit (TaKaRa, Dalian, China, www.takara.com.cn). SYBR Premix Ex Tag Kit (TaKaRa, Dalian, China) was used to perform real-time PCR. A two-stage RT-PCR amplification reaction was performed under the following conditions: 95°C for 30 s, followed by 40 cycles at 95°C for 5 s, and at 60°C for 30 s. The primer sequences were: AQP4 forward primer: 5′-GGGTTGGACCAATCATAGG CG-3′, reverse primer: 5′-GCAGGAAATCTGAGGCCAGTTCTAGG-3′; MMP-9 forward primer: 5′-CGCTGACAAGAAGTGGGGTTT−3′, reverse primer: 5′-TACAGATGGTGGATGCCTTTTA G−3′; GAPDH forward primer: 5′-TGAACGGGAAGCTCACTGG−3′, reverse primer: 5′-GCTTCACCACCTTCTTGATGTC-3′.

### Gelatin Zymography

Previous zymography protocol was followed here (Cai et al., 2015). Briefly, 50 μg samples were diluted in the zymogram sample buffer (Bio-Rad) and electrophoresed using SDS-PAGE for ~2.5 h. Then the gels were incubated four times in renaturing buffer (2.5% Triton X- 100, 50 mmol/L Tris-HCl) for 15 min each with gentle shaking, and then moved to developing buffer for 30 min with gentle shaking at room temperature. Next, gels were stained with Coomassie Blue (0.05% Coomassie Brilliant Blue, 30% methanol, 10% acetic acid) for 3 h after incubation in fresh developing buffer for 3 days at 37°C, and then 30% methanol containing 10% acetic acid was used for de-staining to achieve proper color contrast. The final bands were quantified using the Image J software (NIH, Bethesda, MD, USA, RRID: SCR_003070).

### Statistical Analysis

Data are shown as mean ± SD. Statistical analysis was performed using SPSS and GraphPad PRISM 6.0 software (RRID: SCR_002798) for both parametric and non-parametric comparisons. Unpaired two-tailed Student's *t*-test (between two groups) and one-way ANOVA followed by Student–Newman–Keuls (among multiple groups) were used to evaluate statistical significance. Differences with *p* < 0.05 were considered significant.

## Results

### NBP Attenuated Brain Edema, Increased Cerebral Blood Flow, and Improved Neurobehavioral Outcomes in Transient Middle Cerebral Artery Occlusion Rats

**Figure 2:**
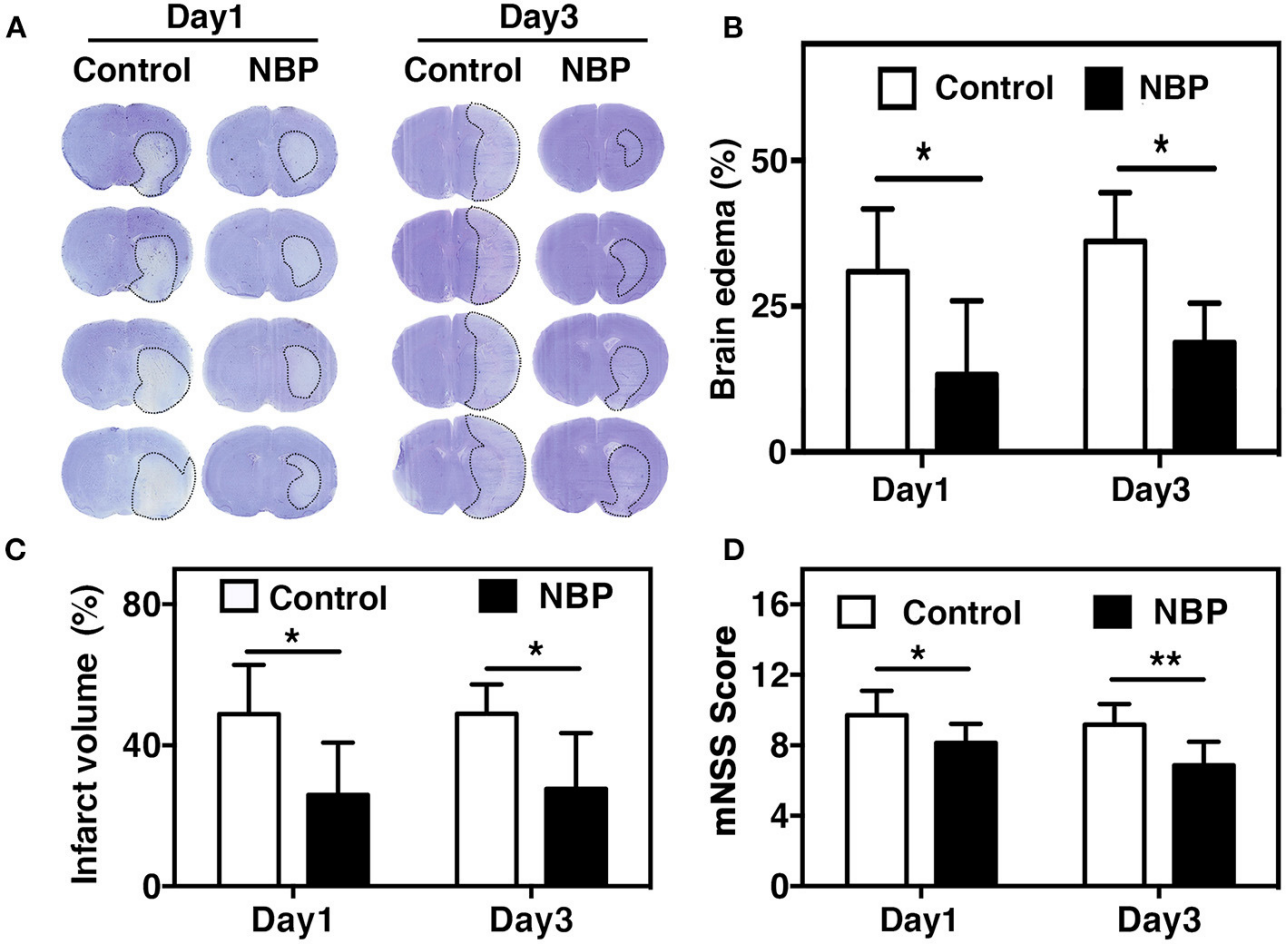
NBP reduced edema and improved neurobehavioral outcomes in tMCAO rats. **(A)** Photomicrography represents the cresyl violet staining in NBP-treated and the control rats at 1 and 3 days after tMCAO. The dashed line indicates the border of the infarct area. Semi-quantification of the brain edema volume **(B)** and the infarct volume **(C)** in the NBP-treated and the control rats at 1 and 3 days after tMCAO. (**D)** Bar graph shows the mNSS assessment in the NBP-treated and the control rats at 1 and 3 days after tMCAO. Data are mean ± SD; *n* = 8 per group; *p < 0.05 and **p < 0.01; NBP-treated rats vs. control rats.

Blood parameters including pH, *P*CO_2_, *P*O_2_, sodium, potassium, and glucose concentration were measured before the surgery, 10 min after occlusion, and immediately after reperfusion. No significant changes in physiological parameters were detected before and after tMCAO operation (Table 1). Since ischemia-induced BBB disruption could lead to brain edema, we measured brain edema and brain infarct volume in NBP-treated rats at 1 and 3 days after tMCAO. The results showed that the ischemic infarct area decreased in the NBP-treated rats compared to that of the control (Figure 2A). It was noted that the infarct area was limited to the striatum in the NBP-treated rats, while it was larger in both the cortex and striatum of control rats. NBP treatment also decreased the brain edema volume and infarct volume (Figures 2B,C) compared to the control after 1 and 3 days of tMCAO (*p* < 0.05). Meanwhile, the mNSS evaluation showed that NBP treatment reduced neurobehavioral deficiency after ischemic brain injury both at 1 and 3 days of tMCAO (*p* < 0.01, Figure 2D).

**Table 1:** Analysis of blood parameters.

	**Baseline**		**MCAO**		**Reperfusion**		
	**Oil**	**NBP**	**Oil**	**NBP**	**Oil**	**NBP**	
sO_2_	95.5 ± 0.5	95.1 ± 0.3	96.5 ± 0.2	95.2 ± 0.3	96.1 ± 0.9	95.6 ± 0.8	95–98%
*P*CO_2_	51.4 ± 1.7	49.2 ± 1.2	42.8 ± 1.5	42.4 ± 1.1	46.2 ± 1.3	46.7 ± 1.7	41–51 mmHg
pH	7.35 ± 0.01	7.34 ± 0.01	7.40 ± 0.02	7.37 ± 0.01	7.37 ± 0.01	7.32 ± 0.02	7.31–7.41
*P*O_2_	82 ± 0.38	82 ± 0.38	92.57 ± 3.21	92.57 ± 3.21	92.57 ± 2.89	92.57 ± 2.89	80–105 mmHg
Na	139.75 ± 0.75	138.57 ± 0.61	138.63 ± 0.63	138.71 ± 0.84	138.13 ± 0.77	139.5 ± 0.76	138–146
K	4.83 ± 0.12	4.84 ± 0.18	4.83 ± 0.17	4.89 ± 0.14	4.83 ± 0.23	4.74 ± 0.34	3.5–4.9
Glu	92.25 ± 8.11	97.0 ± 15.16	99.63 ± 6.31	93.20 ± 5.32	96.43 ± 8.69	90.86 ± 8.69	70–105

*Blood parameters (sO_2_, pO_2_, pCO_2_, K, Na, Glu) before and during the occlusion, and after reperfusion. Data are mean ± SD; n = 8 per group*.

**Figure 3:**
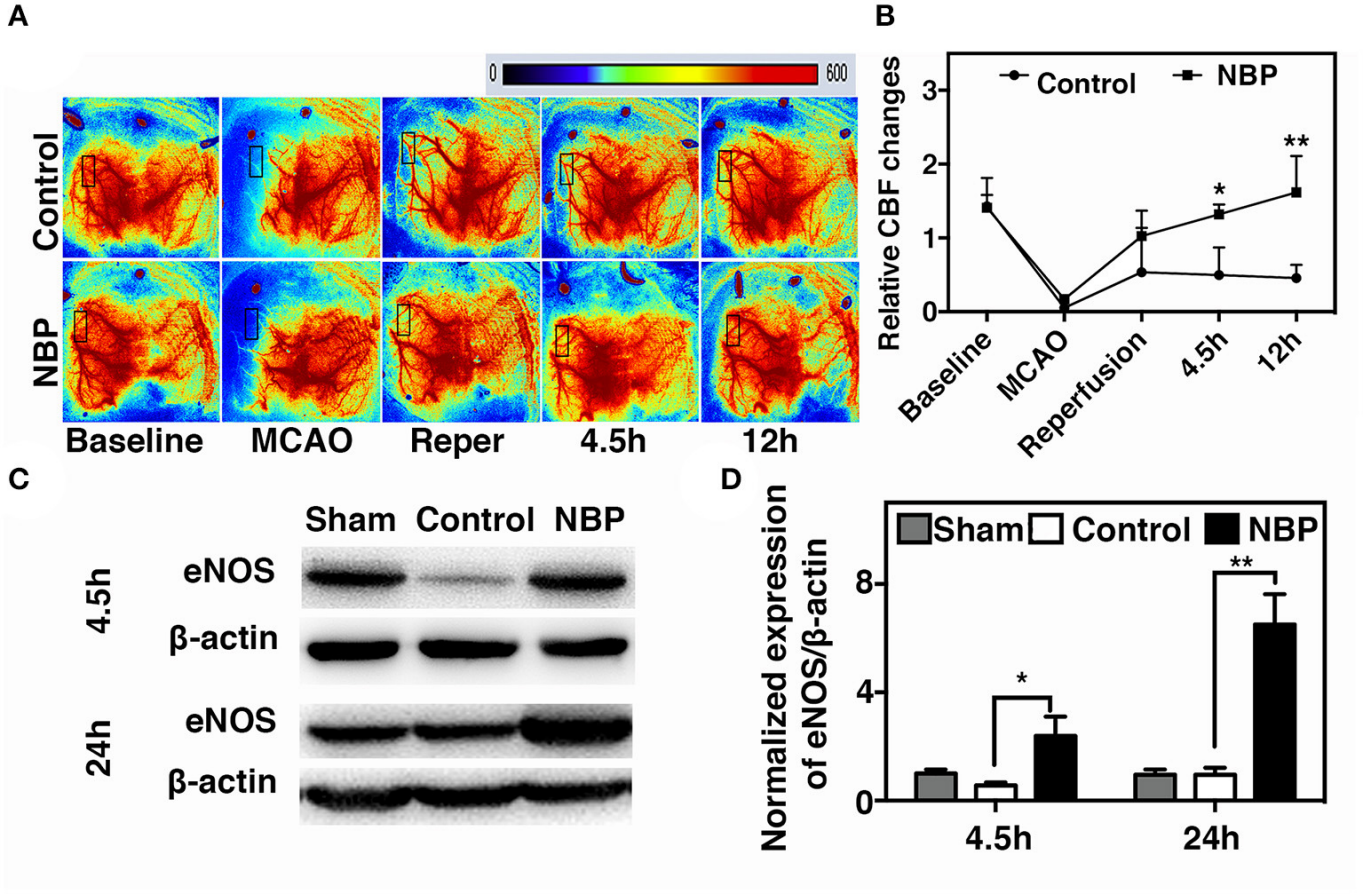
NBP increased cerebral blood flow (CBF) in rats after tMCAO. **(A)** Photomicrographs show CBF measured by Laser speckle contrast imaging (LSCI) at the baseline, 10 min after MCAO, 10 min after reperfusion, and 4.5 and 12 h after occlusion in NBP-treated and control animals. **(B)** Bar graph shows the change in surface CBF in the ipsilateral hemisphere at different time points. Data are mean ± SD; *n* = 5 per group; *p < 0.05 and **p < 0.01; NBP-treated group vs. control animals. Bar graphs show Western blot images **(C)** and eNOS protein expression **(D)** in the NBP-treated and the control rats at 2.5 and 24 h after reperfusion. Data are mean ± SD; *n* = 3 per group; *p < 0.05 and **p < 0.01; NBP-treated group vs. control animals.

A laser speckle contrast imaging system was used to monitor CBF during tMCAO surgery and assess the CBF dynamic changes after NBP treatment. The results showed that NBP treatment significantly increased CBF after reperfusion (*p* < 0.05) and maintained the increase until 12 h after tMCAO (*p* < 0.01). Meanwhile, the control group showed a decreasing tendency in CBF changes after reperfusion (Figures 3A,B). To further explore which molecular mechanism induces increase in CBF, we examined endothelial nitric oxide synthase (eNOS) expression due to its role in vasodilation (Coletta et al., 2012). Our results showed that NBP treatment increased eNOS protein expression compared to the control at 4.5 and 24 h after occlusion (Figures 3C,D, *p* < 0.05), which could explain why CBF increased after NBP treatment.

### DL-3n-Butylphthalide Protected Blood–Brain Barrier Integrity After Transient Middle Cerebral Artery Occlusion

**Figure 4:**
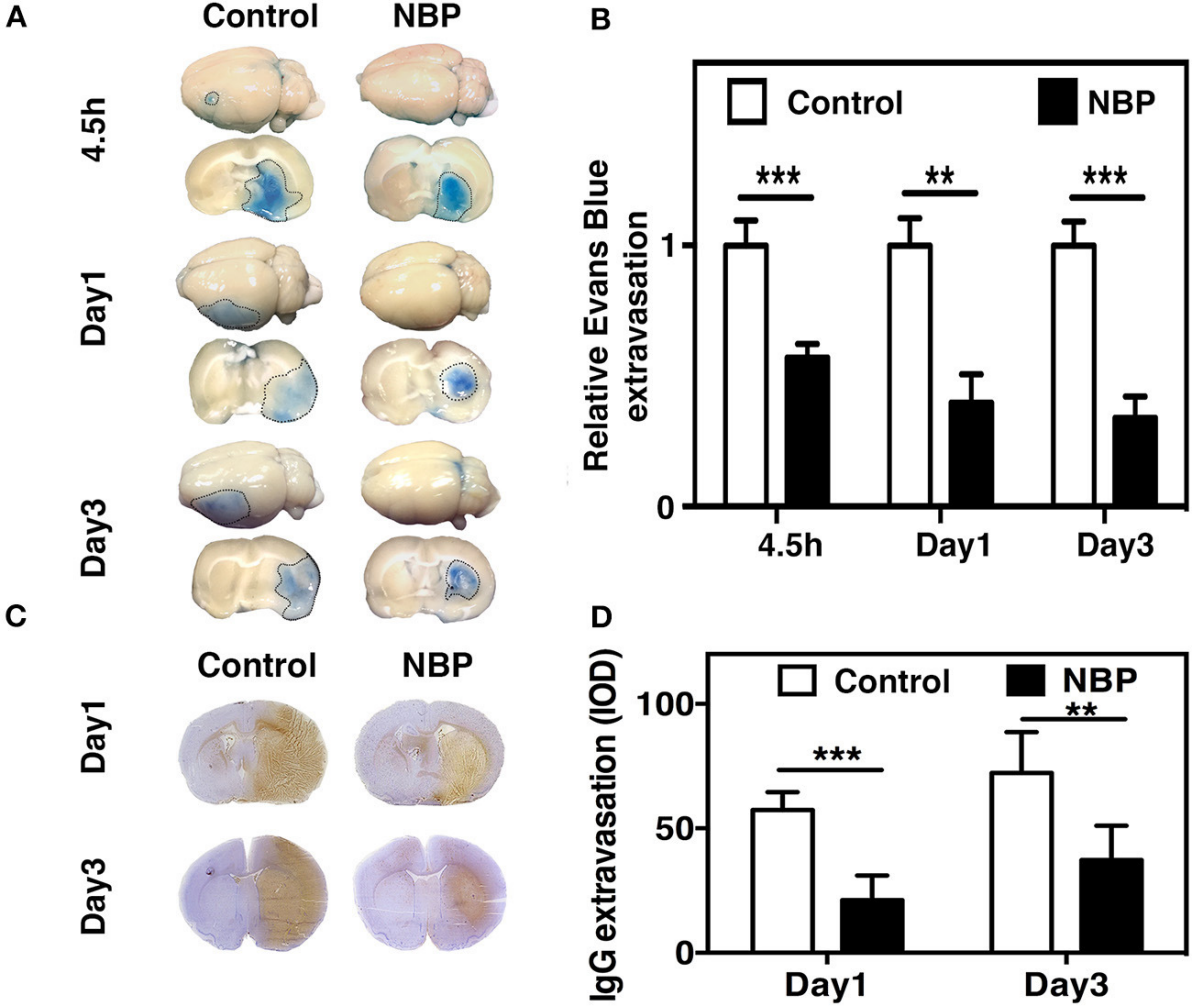
NBP reduced BBB leakage in rats after tMCAO. **(A)** Photographs shows Evans blue (EB) exudation for the whole brain and brain sections in the NBP-treated and control rats at 1 and 3 days after tMCAO. The value of extravasated EB was recorded by a spectrophotometer at 610 nm (g). **(B)** Bar graph shows semi-quantification for the relative EB extravasation in the NBP-treated and the control rats at 4.5 h, 1 day, and 3 days after tMCAO. Data are mean ± SD; *n* = 8 per group; **p < 0.01 and ***p < 0.001; NBP-treated rats vs. control rats. **(C)** Photographs show the IgG leakage in the ipsilateral hemisphere of NBP-treated and control rats following 1 and 3 days after tMCAO. **(D)** Bar graph shows semi-quantification of IgG extravasation in the NBP-treated and control rats. Data are mean ± SD; *n* = 7 per group; **p < 0.01, and ***p < 0.001; NBP-treated rats vs. the control rats.

**Figure 5:**
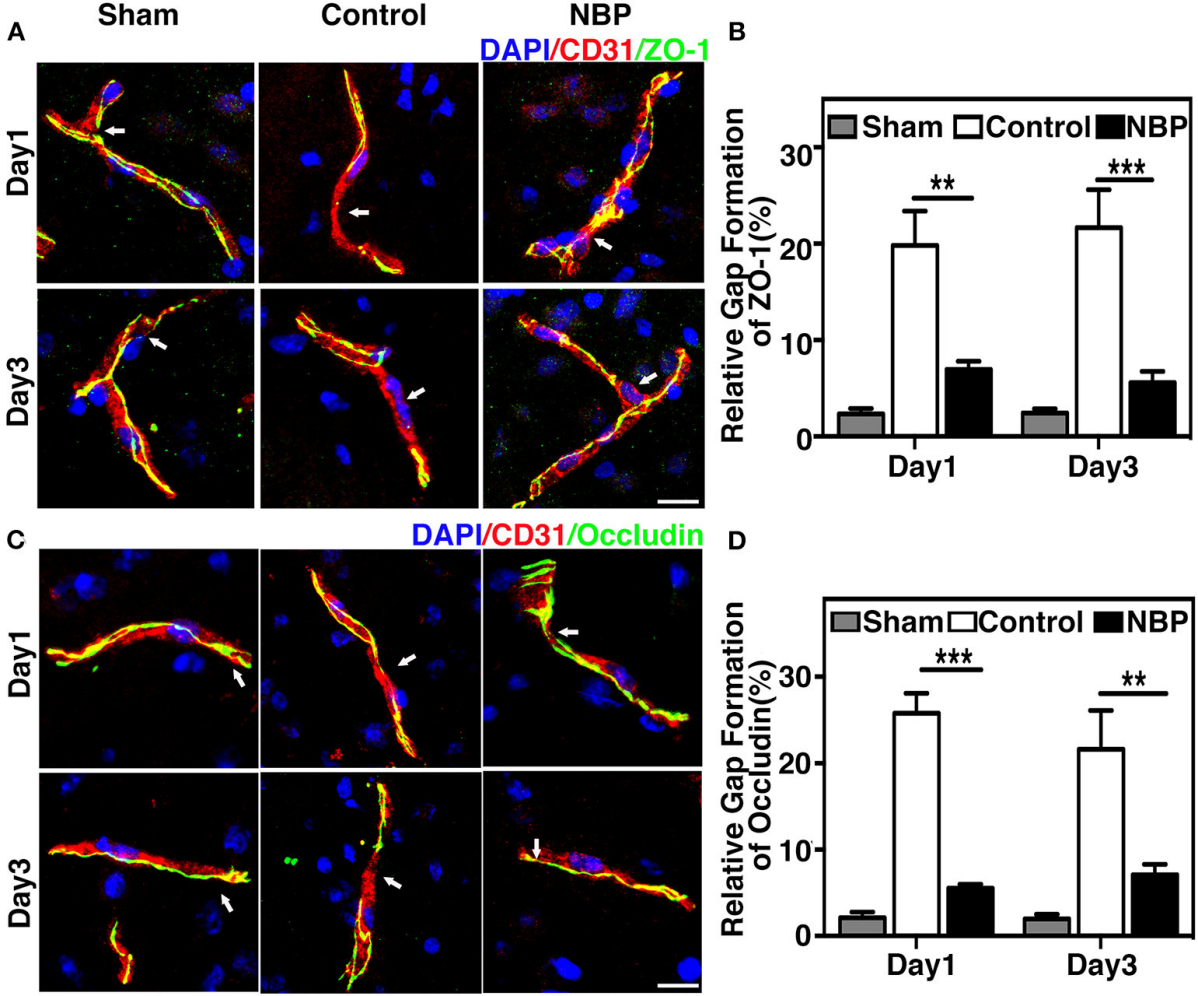
NBP reduced BBB tight junction disruption in rats after tMCAO. **(A)** Photomicrographs show the ZO-1 (green) and CD31 (red) double immunostaining in the NBP treated and control rats at 1 and 3 days after tMCAO. Scale bar = 25 μm. **(B)** Bar graph shows the semi-quantification of gap length for ZO-1-positive staining. **(C)** Photomicrographs showed the occludin (green) and CD31 (red) double immunostaining in the NBP-treated and control rats at 1 and 3 days after tMCAO. Scale bar = 25 μm. **(D)** Bar graph shows the semi-quantification of gap length for occludin-positive staining. Data are mean ± SD; *n* = 5 per group; **p < 0.01, ***p < 0.001; NBP-treated rats vs. the control rats.

To evaluate BBB functional integrity, we injected 2% Evans blue (EB) in the rats using the jugular vein on 4.5 h, day 1, and day 3 after tMCAO. EB extravasation was limited to the striatum of the ipsilateral hemisphere after NBP treatment, while EB extravasation was detected in both the striatum and cortex in the control group (Figure 4A), which was consistent with the cresyl violet staining results (Figure 2A). The quantification of EB results showed that the total amount of extravasated EB decreased in NBP-treated rats compared to the control at 1 and 3 days after tMCAO (*p* < 0.01, *p* < 0.001, Figure 4B). To further evaluate BBB permeability, we stained the brain sections with the IgG antibody to assess the severity of the IgG invasion at 1 and 3 days after tMCAO. Results showed that IgG invasion mainly occurred in the striatum of NBP-treated rat brains, while both striatum and cortex showed IgG invasion in the control (Figure 4C). Semi-quantification analyses showed that NBP treatment decreased IgG invasion compared to the control at 1 and 3 days after tMCAO (*p* < 0.01, *p* < 0.05, Figure 4D). Furthermore, to investigate whether NBP protected BBB structural integrity, we stained the tight junction proteins ZO-1 and occludin with endothelial cell marker CD31. The confocal microscopy images showed that NBP treatment significantly attenuated ZO-1 and occludin-mediated gap formation in the microvessel wall induced by stroke, thereby preserving tight junction protein structural integrity at 1 and 3 days after tMCAO (Figures 5A–D).

### DL-3n-Butylphthalide Preserved Blood–Brain Barrier Integrity by Decreasing AQP4 Expression and MMP-9 Activity After Transient Middle Cerebral Artery Occlusion

**Figure 6:**
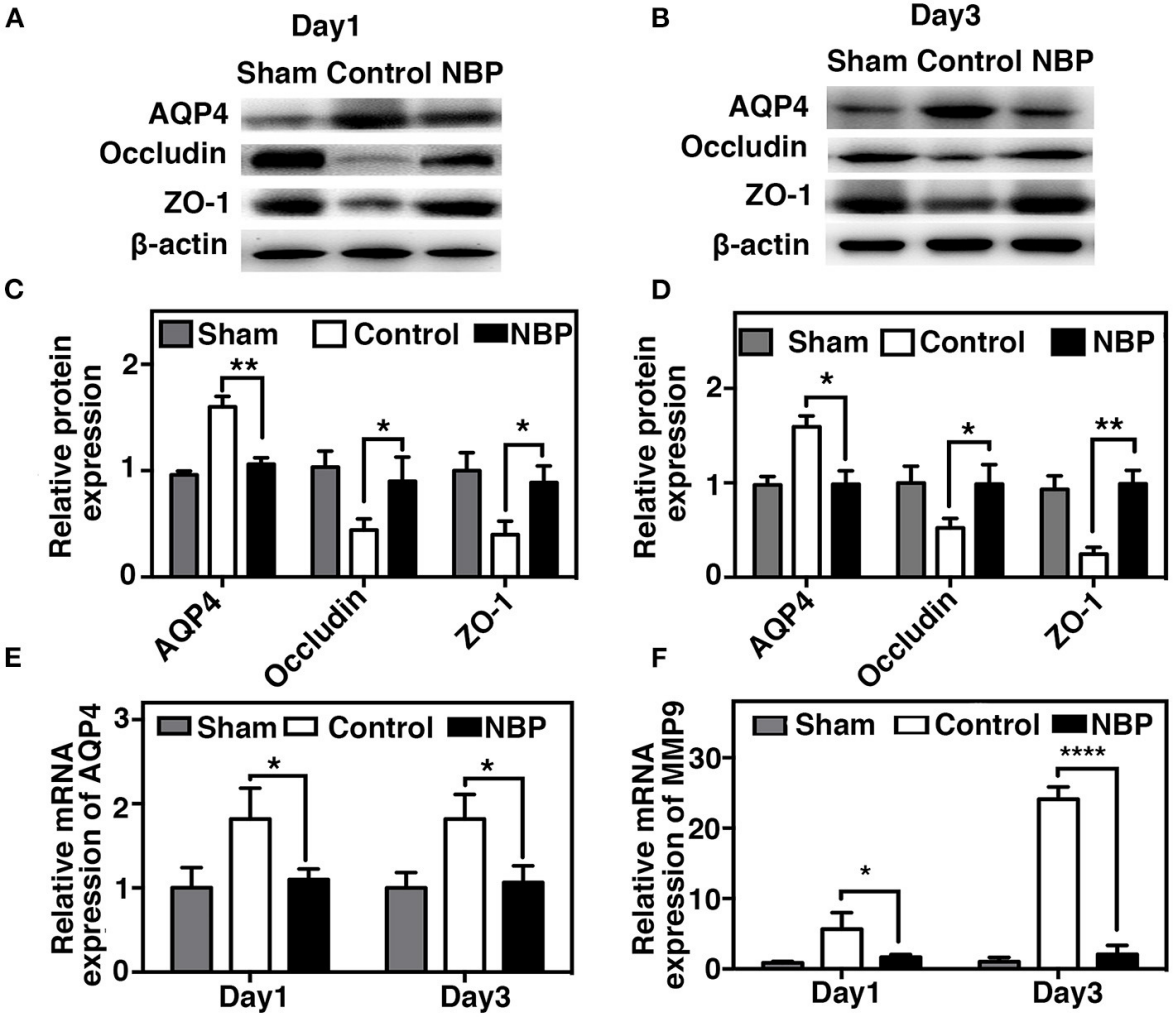
NBP decreased AQP4 expression and tight junction protein loss after tMCAO. Photomicrographs showing the Western blot of AQP4, ZO-1, and occludin expression in the NBP-treated and control rats at 1 **(A)** and 3 days **(B)** after tMCAO. Bar graphs show the semi-quantification of AQP4, ZO-1, and occludin expression in the NBP-treated and control rats at 1 day **(C)** and 3 days **(D)** after tMCAO, and relative AQP4 **(E)** and matrix metalloproteinase-9 (MMP-9) **(F)** mRNA expression in the NBP-treated and control rats. Data are presented as mean ± SD; *n* = 3 per group; *p < 0.05, **p < 0.01, ****p < 0.0001; NBP-treated rats vs. control rats.

AQP4 overexpression and disruption of ionic balance contribute to brain edema after ischemic brain injury (Yang et al., 2007; Kleffner et al., 2008). Our results demonstrated that NBP could downregulate AQP4 expression at both the protein (*p* < 0.01, *p* < 0.05, Figures 6A–D) and mRNA levels (*p* < 0.01, Figure 6E). Occludin expression increased in NBP-treated rats at 1 and 3 days after tMCAO, while ZO-1 expression only increased at 3 days after tMCAO (Figures 6A–D, *p* < 0.05).

**Figure 7:**
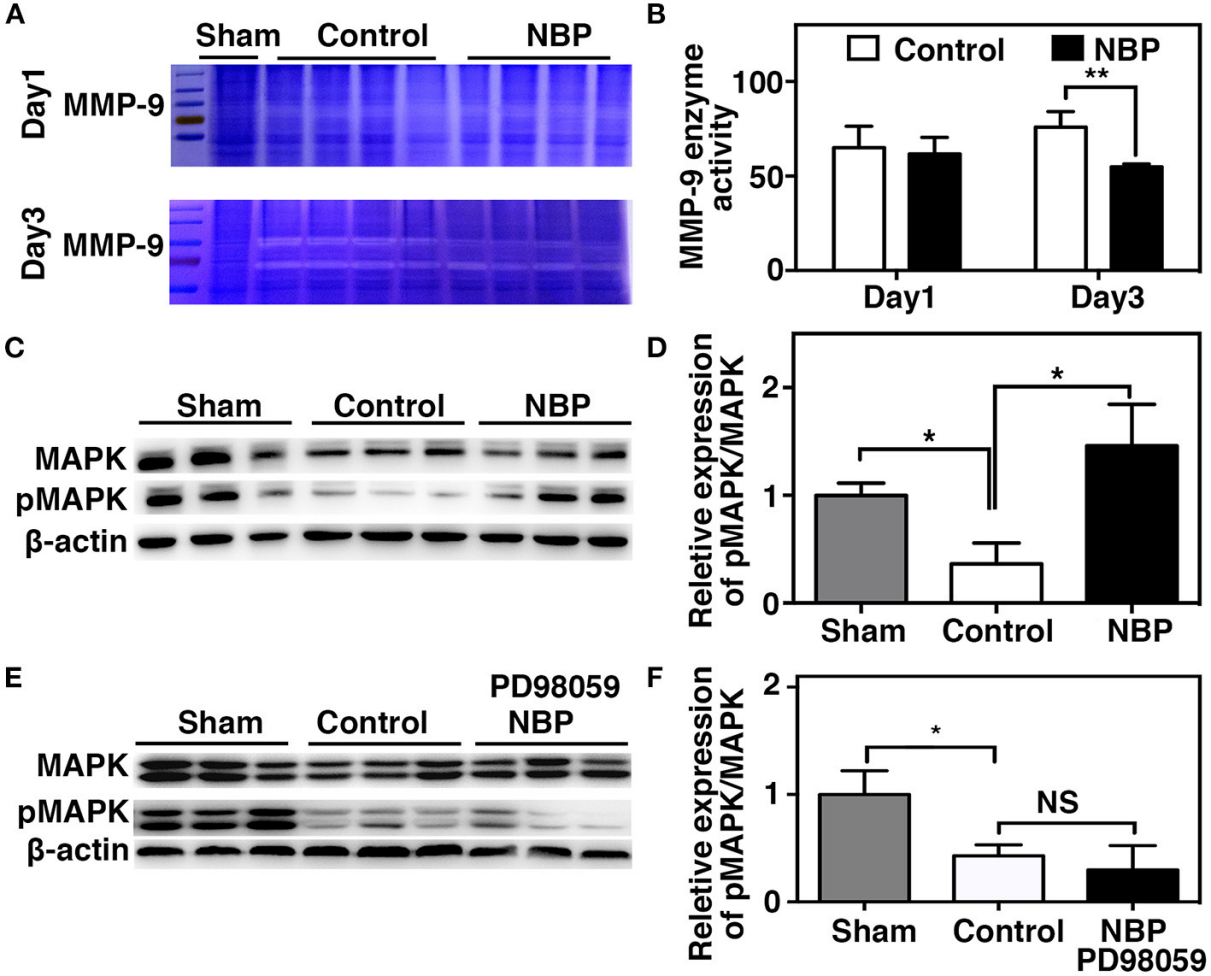
NBP decreased MMP-9 enzyme activity, increased MAP kinases phosphorylation after tMCAO. **(A)** Photomicrograph shows zymography in NBP-treated and control rats at 1 and 3 days after tMCAO. **(B)** Bar graph shows the semi-quantification of active MMP-9 levels. Data are mean ± SD; *n* = 4 per group; **p < 0.01; NBP-treated rats vs. control rats. **(C)** Photomicrograph showed a Western blot of MAP kinases phosphorylation in the NBP-treated, control, and sham rats. **(D)** Bar graph shows semi-quantification of phospho-MAP kinase at 3 days after tMCAO. **(E)** Photomicrograph showed a Western blot of MAP kinases phosphorylation in the sham, Control, and PD98059+NBP-treated rats. **(F)** Bar graph shows semi-quantification of phospho-MAP kinase at 3 days after tMCAO. Data are mean ± SD, *n* = 3 per group; *p < 0.05, **p < 0.01; NBP-treated rats vs. control rats.

MMP-9 mediates BBB permeability by degrading tight junction proteins in the acute stage of stroke (Yang et al., 2007; Rosell et al., 2008). We next examined whether NBP had a protective effect on BBB permeability by reducing MMP-9 activity after ischemic brain injury. First, we found that MMP-9 at the mRNA levels decreased in the NBP-treated rats compared to the control after 1 and 3 days after tMCAO (*p* < 0.05, Figure 6F). Second, to evaluate the MMP-9 enzyme activity, gelatin zymography was performed in the NBP-treated and control rats at 1 and 3 days after tMCAO. Results showed that MMP-9 activity was reduced in the NBP-treated rats compared to the control rats at 3 days after tMCAO (*p* < 0.01, Figures 7A,B).

### MAPK Signaling Pathway Involved in the Protective Role of DL-3n-Butylphthalide After Transient Middle Cerebral Artery Occlusion

To further explore the signaling pathway involved in the protective role of NBP on BBB integrity after tMCAO, we examined the MAPK signaling pathway. The results showed that MAP kinase phosphorylation decreased in the NBP-treated rats compared to the control at 3 days after tMCAO (Figures 7C,D, *p* < 0.05). PD98059 inhibited MAPK phosphorylation in PD98059+NBP-treated group compared to the control group after tMCAO and diminished the effect of NBP on MAPK phosphorylation (Figures 7E,F, *p* < 0.05).

## Discussion

**Figure 8:**
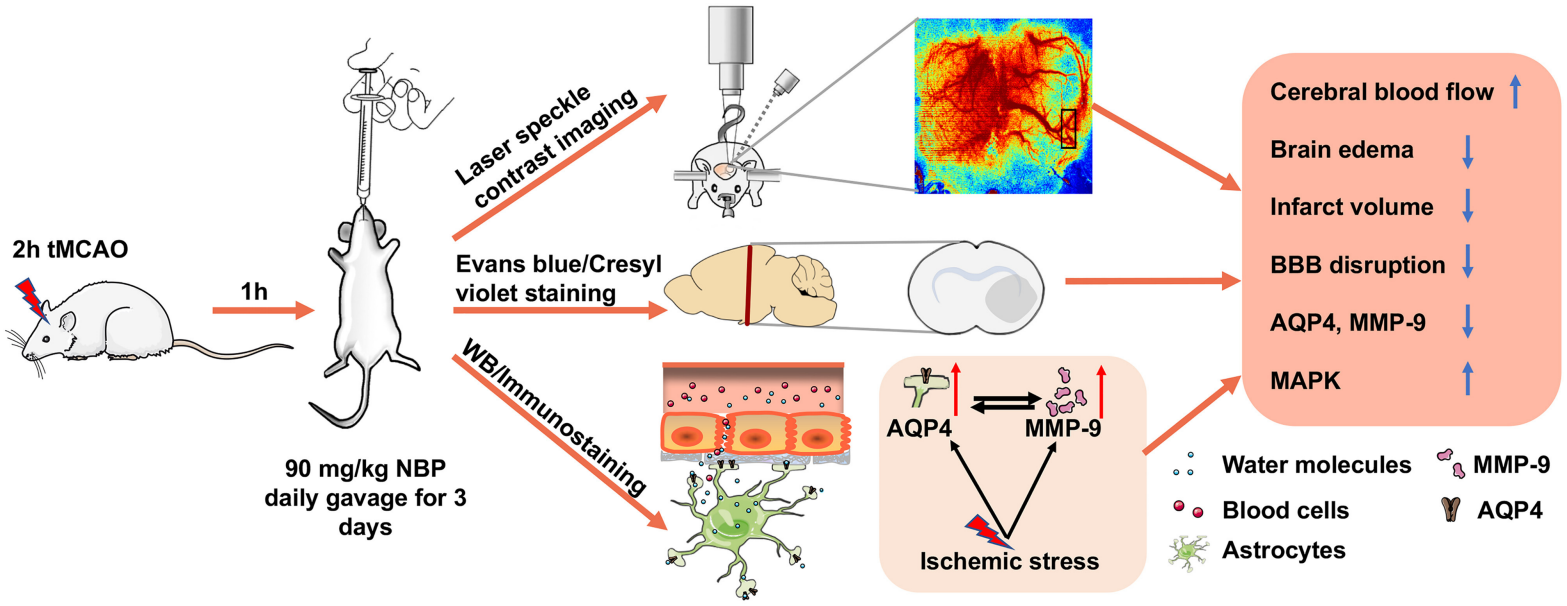
Therapeutic effect of NBP during tMCAO. Diagram illustrates that adult SD rats underwent tMCAO and were treated with NBP for 3 days. The results demonstrated that NBP treatment could increase CBF of the ipsilateral hemisphere, attenuate infarct volume and brain edema, reduce BBB disruption, and inhibit ischemia-induced increase in AQP4 and MMP-9 expression, which is associated with MAP kinase phosphorylation.

In the present study, we demonstrated that NBP (1) attenuated ischemia-induced brain edema and neuronal death; (2) improved neurological function recovery; (3) inhibited AQP4 expression in the ischemic brain and reduced tight junction protein loss; (4) and partially inhibited MMP-9 expression and activity (5) through the possible MAPK signaling pathway associated in BBB disruption (Figure 8). Our results provide experimental evidence that NBP improves the damaged BBB integrity during brain edema, suggesting that NBP could be used for BBB disruption induced by ischemia or other brain diseases.

Ischemic brain injury is common; it causes neuronal apoptosis, inflammatory response, free radical oxidants, and BBB disruption (Feigin et al., 2017). The disrupted BBB allows vasculature-derived substances to infiltrate the brain, induces secondary brain injury and edema, and aggravates ischemic stroke outcomes (Sandoval and Witt, 2008). We chose days 1 and 3 after tMCAO because BBB damage occurs in the early stage after stroke (Rosenberg, 1999; Khatri et al., 2012). We present the data at days 1 and 3 to interpret the effect of NBP on BBB integrity after ischemia. NBP has been used in the acute phase after ischemic stroke after several studies demonstrated its neuroprotective effects including dilation of blood vessels, promotion of angiogenesis, suppression of inflammation, antioxidative stress, protection of the structure and function of mitochondria, and inhibition of thrombosis (Abdoulaye and Guo, 2016; Qin et al., 2019; Yang et al., 2019; Zhou et al., 2019). NBP also attenuated ischemic brain injury by decreasing infarct volume, neuronal apoptosis, and neurological deficits in the peri-infarct areas in a rat disease model (Li et al., 2010; Zhang et al., 2012a,b). Clinical and experimental studies showed that as a lipid-soluble drug, NBP could directly pass through the BBB and exert its protective effects on the brain (Liu et al., 2007; Cao et al., 2009; Liao et al., 2009; Wang et al., 2011; Zhang et al., 2012a, 2017). *In vitro* study showed that NBP could protect BBB tight junction protein by decreasing the endothelial intracellular ROS generation (Ye et al., 2019). Our results demonstrated that NBP had protective effects on the BBB in an acute ischemic stroke animal model. The results showed that NBP reduced tight junction protein loss and AQP4 expression. Since the inhibition of AQP4 protein ameliorated ischemic brain injury by reducing early cytotoxic brain edema, NBP may further protect the BBB from ischemia and reperfusion injury by downregulating AQP4. Preservation of the BBB tight junction proteins is essential for maintaining BBB integrity, which prevents secondary brain injury and is closely correlated with AQP4 downregulation (Tang et al., 2014; Filchenko et al., 2020). NBP has a therapeutic effect on the disrupted BBB in the early phase of ischemic stroke.

MMP-9 also plays a critical role in maintaining BBB integrity (Cai et al., 2015). Studies have shown that MMP-9 is not only involved in the pathogenesis of BBB disruption and subsequent vasogenic edema following stroke but also in hemorrhagic transformation (Lakhan et al., 2013; Shi et al., 2016). MMP-9 upregulation results in the degradation of tight junction proteins such as ZO-1, occludin, and the basal lamina, ultimately triggering BBB disruption and brain edema in the acute stage of stroke (Yang et al., 2007; Lee et al., 2013; Mamtilahun et al., 2019). The inhibition of MMP-9 could reverse this effect (Wang et al., 2012). Here, we found that NBP could inhibit MMP-9 mRNA expression and MMP-9 enzyme activity. NBP-mediated MMP-9 activity downregulation could reduce BBB tight junction protein degradation and protect BBB integrity.

Additionally, we found that NBP increased MAP kinase phosphorylation. Studies have shown that MAP kinase ERK1/2 modulates the internalization and degradation of tight junction proteins claudin-2, claudin-4, occludin, and ZO-1 (Rincon-Heredia et al., 2014; Stamatovic et al., 2017). Phosphorylation of MAPK is usually induced by pro-inflammatory stimuli; it also can be activated by growth factor (Sun and Nan, 2016). In the present study, we found that NBP treatment modulated the MAPK phosphorylation to the same level as the sham group after tMCAO, and treating with MAPK inhibitor reversed the result. Previous studies showed that NBP treatment upregulates the VEGF and basic fibroblast growth factor (bFGF) expressions in ischemic stroke patients and improve neurobehavioral recovery in rat ischemic stroke model (Liao et al., 2009; Tang et al., 2017; Zhou et al., 2019); growth factor binds to its receptor to activate MAPK by B-Raf (Kao et al., 2001). Taken together, the increased phosphorylation of MAPK may be due to the upregulated growth factor after NBP treatment, which is closely related to angiogenesis and long-term recovery after stroke. Our results support that NBP protects BBB integrity by increasing the phosphorylation of MAP kinase pathway after stroke.

## Conclusion

Our study demonstrated that NBP treatment increased CBF and preserved BBB integrity by inhibiting AQP4 and MMP-9 enzyme activity. MAP kinases signaling pathways are possibly associated in this mechanism.

## Data Availability Statement

The original contributions presented in the study are included in the article/supplementary materials, further inquiries can be directed to the corresponding authors.

## Ethics Statement

The animal surgery and experimental protocol were approved by the Institutional Animal Care and Use Committee (IACUC), Shanghai Jiao Tong University, Shanghai, China.

## Author Contributions

MM participated in the research design, all experimental procedures, animal surgery, data analysis, and drafting of the first manuscript. ZW and CQ contributed to animal surgery, behavioral tests, and data collection. YT provided technical assistance for *in vivo* and *in vitro* experiments. H-LT and YW were involved in discussion of the research design, the results, and edited the manuscript. G-YY and ZZ supervised all aspects including research design, data analysis, and manuscript preparation. All authors read and agreed to the final manuscript.

## Conflict of Interest

The authors declare that the research was conducted in the absence of any commercial or financial relationships that could be construed as a potential conflict of interest.

## Abbreviations

AQP4: Aquaporin4
BBB: blood–brain barrier
EB: Evans blue
eNOS: endothelial nitric oxide synthase
LSCI: Laser speckle contrast imaging
MCAO: middle cerebral artery occlusion
MMP-9: matrix metalloproteinase-9
NBP: dl-3n-butylphthalide
mNSS: modified neurological severity scores
ROS: reactive oxygen species
CBF: cerebral blood flow
PFA: paraformaldehyde
ZO-1: zonula occludens-1.
